# Diversity and Plant Growth-Promoting Effects of Fungal Endophytes Isolated from Salt-Tolerant Plants

**DOI:** 10.4014/jmb.2006.06050

**Published:** 2020-08-31

**Authors:** Irina Khalmuratova, Doo-Ho Choi, Ju-Ri Woo, Min-Ji Jeong, Yoosun Oh, Young-Guk Kim, In-Jung Lee, Yeon-Sik Choo, Jong-Guk Kim

**Affiliations:** 1 School of Life Science and Biotechnology, Kyungpook National University, Daegu 4566, Republic of Korea; 2 School of Applied Biosciences, Kyungpook National University, Daegu 41566, Republic of Korea; 3 Department of Biology, College of National Sciences, Kyungpook National University, Daeagu 41566, Republic of Korea

**Keywords:** Halophytic plants, endophytic fungi, diversity, salt marsh, plant growth promotion, gibberellins

## Abstract

Fungal endophytes are symbiotic microorganisms that are often found in asymptomatic plants. This study describes the genetic diversity of the fungal endophytes isolated from the roots of plants sampled from the west coast of Korea. Five halophytic plant species, *Limonium tetragonum*, *Suaeda australis*, *Suaeda maritima*, *Suaeda glauca Bunge*, and *Phragmites australis*, were collected from a salt marsh in Gochang and used to isolate and identify culturable, root-associated endophytic fungi. The fungal internal transcribed spacer (ITS) region ITS1-5.8S-ITS2 was used as the DNA barcode for the classification of these specimens. In total, 156 isolates of the fungal strains were identified and categorized into 23 genera and two phyla (Ascomycota and Basidiomycota), with Dothideomycetes and Sordariomycetes as the predominant classes. The genus *Alternaria* accounted for the largest number of strains, followed by *Cladosporium* and *Fusarium*. The highest diversity index was obtained from the endophytic fungal group associated with the plant *P. australis*. Waito-C rice seedlings were treated with the fungal culture filtrates to analyze their plant growth-promoting capacity. A bioassay of the Sm-3-7-5 fungal strain isolated from *S. maritima* confirmed that it had the highest plant growth-promoting capacity. Molecular identification of the Sm-3-7-5 strain revealed that it belongs to *Alternaria* alternata and is a producer of gibberellins. These findings provided a fundamental basis for understanding the symbiotic interactions between plants and fungi.

## Introduction

Unique microecosystems within the internal tissues of plants support diverse, symbiotic microbial communities that greatly influence plant adaptation and evolution [1, 2]. These microbial associations significantly influence the ability of plants to adapt and evolve in their environment. All plants in natural ecosystems appear to develop symbiotic associations with fungi [3, 4]. These symbioses provide a buffer against biotic and abiotic stresses and are important for the survival of plant hosts and their fungal symbionts in high-stress habitats [3, 4]. Both plant and fungi obtain multiple benefits from symbiotic interactions in terms of fitness. Fungal symbionts may impart beneficial (mutualism, commensalism, and parasitism), harmful (competition), or neutral (amensalism and neutralism) activities on the plant host. Concurrently, host plants can also interact beneficially (mutualism), neutrally (commensalism and neutralism), or harmfully (parasitism, competition, and amensalism) during symbiosis with the fungi [5-7].

Microbes living within plants are referred to as ‘endophytes’ and are host-specific fungi or bacteria, such as Actinomycetes spp. [8, 9]. Endophytes constitute a major portion of the fungal symbionts associated with the roots, stems, and leaves of plants, and generally do not impart adverse effects to the host [10, 11]. They comprise a diverse group and represent a wide taxonomical range of fungi belonging primarily to the phylum Ascomycota [12, 13]. Some fungal endophytes can synthesize plant growth hormones such as auxin, abscisic acid, and gibberellins (GAs) [14, 15]. Endophytic fungi increase the host’s resistance to biotic stresses caused by insects, pests, and fungal pathogens and improve the host plant’s fitness against harsh environmental factors [16].

A large percentage of the world’s biodiversity is concentrated within the coastal regions, although much of it is yet to be discovered. The coastal zone is the interface between the land and sea and is characterized by inter-connections among neighboring ecosystems. Coastal salt marsh wetlands, located in this transition zone between land and salt or brackish water, are among the most important and biologically productive natural ecosystems on Earth,. These coastal wetlands provide several ecosystem services, such as nutrient removal, storm protection, and carbon sequestration. However, the harsh coastal environment can also inhibit plant growth due to tidal submergence, anaerobic soil (little or no oxygen present), and high salinity [17, 18]. Moreover, anthropogenic activities contribute significantly to the destruction of salt marsh habitats [19, 20].

This study sought to isolate and identify members of the endophytic fungal community from the roots of halophytic plants, and provide analysis of their diversity and distribution. This work also uses a bioassay of the endophytic fungal isolates applied to Waito-C rice seedlings to observe the effects of plant growth promotion and GA production.

## Materials and Methods

### Collection of Plant Samples

Samples from five mature and healthy plant species, *Limonium tetragonum*, *Suaeda australis*, *Suaeda maritima*, *Suaeda glauca Bunge*, and *Phragmites australis*, from the Gochang salt marsh located on the west coast of Korea, were collected for this study. The sampling sites and scientific names of the five halophytes are listed in Table 1. All samples were immediately placed individually in sterile plastic bags and stored at 4°C upon arrival at the laboratory. Plants were processed within 48 h of being sampled.

### Surface Sterilization and Isolation of Endophytic Fungi from Root Samples

Prior to sterilization, the root samples were thoroughly washed in running tap water to remove sand particles, soil, and other debris. The roots were subjected to a three-step surface sterilization procedure, treated with Tween 80 solution (200 μl in 100 ml distilled water) for 10 min and twice with 1% (w/v) perchloric acid solution for 10 min, followed by washing with distilled water [21-24]. Following these preprocessing steps, the roots were aseptically sectioned into 1.5–2-cm-long fragments. Finally, the plant fragments were transferred onto the isolation media (Hagem minimal medium: 0.5% glucose, 0.05% KH_2_PO_4_, 0.05% MgSO_4_·7H_2_O, 0.05% NH_4_Cl, and 0.1% FeCl3) containing 80 ppm streptomycin and incubated at 25°C [25-27]. All fungi that grew from the inside of the root samples were then transferred onto potato dextrose agar. The isolated pure cultures of root fungi were stored on potato dextrose agar plates and slants [21-23].

### DNA Extraction, PCR Amplification, and Identification of Fungal Strains

The mycelia of the fungal strains were cultivated in potato dextrose broth for 7–10 days at 120 rpm and 25°C. The lyophilized fungal strains were used for identification. The fungal genomic DNA was extracted with a DNeasy Plant Mini Kit (Qiagen, USA) and the internal transcribed spacer (ITS) region of the DNA was amplified using universal primers ITS-1 (5′-TCC GTA GGT GAA CCT GCG G-3′) and ITS-4 (5′-TCC GCT TAT TGA TAT GC-3′). The reaction conditions consisted of an initial denaturation at 95°C for 2 min, followed by 35 cycles of denaturation at 95°C for 30 sec, annealing at 55°C for 1 min, and extension at 72°C for 1 min, and a final extension at 72°C for 7 min. The PCR products were observed by agarose gel electrophoresis with ethidium bromide staining. The products were purified with a QIAGEN QIAquick PCR Purification Kit and sequenced using the ABI PRISM BigDye Terminator Cycle Sequencing Kit (Applied Biosystems, USA) on an ABI 310 DNA sequencer (Perkin-Elmer). The resulting DNA sequence was identified using the Basic Local Alignment Search Tool (BLAST) search program (http://www.ncbi.nlm.gov/BLAST/) from the National Center for Biotechnology Information (NCBI).

### Statistical Analysis

The generic richness and diversity of the fungal endophytes were analyzed at the genus level in the plant samples. Menhinick’s index (*Dmn*) and Margalef ’s index (*Dmg*) were used to determine the richness of each genus in the community [28-29]. The Menhinick’s index was calculated via the following equation: Dmm=S⁄√N ; Margalef ’s index was calculated as follows: Dmg=(S−1)/ln(N), where S is the number of genera in a sample, and N is the total number of individuals in a community. Both indices ranged from 0 to ∞. The genus diversity was evaluated using the Shannon diversity index (*H’*), Fisher’s alpha index ( α), and Simpson’s index of diversity [30-31]. Fisher’s alpha index (α) was calculated as S=α⋅ln(1+N⁄α), where S is the number of genera, and N is the total number of individuals. The equation for Shannon’s diversity index is $H'=-\Sigma_{i=1}^{R}\mathrm{pi}\cdot \mathrm{lnpi}$, where *pi* is the proportion of individuals found in genera *i* in a sample. The values of the Shannon diversity index generally fall between 1.5 and 3.5. Simpson’s index of diversity (*1-D*) was calculated as $D=\Sigma_{i=1}^{R}\mathrm{ni}(\mathrm{ni}–1)⁄N(N–1)$, where *N* is the total number of individuals in a sample, and *ni* is the number of individuals found in genera *i* in a sample. The magnitude of this index ranges between 0 and 1; the greater the magnitude, the greater the sample diversity.

### Effect of Fungal Filtrates for Plant Growth Promotion in Waito-C Rice Seedlings

The culture filtrates of the isolated fungal strains were bioassayed in Waito-C rice seedlings to determine their plant growth-promoting activity. The fungal isolates were grown in a shaking incubator for 7 days at 25°C and 180 rpm in Czapek broth medium (1% glucose, 1% peptone, 0.1% K_2_HPO_4_, 0.05% KCl, 0.05% MgSO_4_·7H_2_O, and 0.001% FeSO_4_·7H_2_O; /L pH 7.3 ± 0.2). Forty-five milliliters of culture fluid was harvested; the pellet and the supernatant were stored at −70°C and then lyophilized. The lyophilized supernatants were mixed with 1 ml of autoclaved distilled water. The Waito-C rice seeds were treated overnight with uniconazole 20 ppm to minimize the activity of the seed coat gibberellins. The treated Waito-C rice seeds were washed and soaked in autoclaved distilled water until the sprouts emerged. Then the Waito-C rice seedlings were transplanted into glass tubes containing a 0.6% water-agar medium and grown in a growth chamber. Ten microliters of the supernatant solution for each fungal culture filtrate was applied to the apical meristem of the Waito-C rice seedlings at the two-leaf stage. One week after treatment, the plant and shoot length were observed and compared with the controls. The controls included culture filtrate of *Gibberella fujikuroi*, Czapek broth medium, and distilled water [30, 32].

### Extraction and Quantification of Gibberellins (GAs) from the Fungal Culture Filtrates

The culture filtrate of each endophytic fungal isolate was analyzed for the presence of GAs by gas chromatography/mass spectrometry (GC/MS). Endophytic fungal isolates were cultured in 250 ml of Czapek broth medium (containing 1% (w/v) glucose and peptone) for 7 days at 25°C in a shaking incubator at 180 rpm. The extracted GAs were analyzed by reverse-phase C18 high-performance liquid chromatography (HPLC). The fractions were collected and prepared for GC/MS via selected ion monitoring (SIM) (6890N Network GC System, 5973 Network Mass Selective Detector, Agilent Technologies, USA). Following the GC/MS, all data were collected and analyzed. The three major ions of the supplemented [^2^H_2_] GAs’ internal standards and the fungal GAs were monitored simultaneously. The retention time was determined using hydrocarbon standards to calculate the Kovats retention index (KRI) value, while quantification of the GAs was based on peak area ratios of the nondeuterated (extracted) GAs to deuterated GAs [31, 33].

## Results and Discussion

### Molecular Identification of the Endophytic Fungi

In total, 156 fungal endophytes, belonging to 26 species and 23 genera, were isolated from the roots of five halophytic plant species native to the Gochang salt marsh. The nucleotide sequence for each strain was submitted to the GenBank database of the National Center for Biotechnology Information (Accession Nos. KP018058-KP018213). The fungal similarity scores obtained were at or close to 100%.

Most of the endophytic fungal strains belonged to the phylum Ascomycota (153/156 strains), while the phylum Basidiomycota (3/156 strains) only represented a minor part of the entire fungal community. The class Dothideomycetes (117/156 strains) accounted for the highest number of strains, followed by the classes Sordariomycetes (26/156 strains), Eurotiomycetes (10/156 strains), Ustilaginomycetes (2/156 strains), and Exobasidiomycetes (1/156 strains). At the genus level, *Alternaria* (52/156) accounted for the largest proportion, followed by *Cladosporium* (39/156), and *Fusarium* (16/156).

**Fig. 1:**
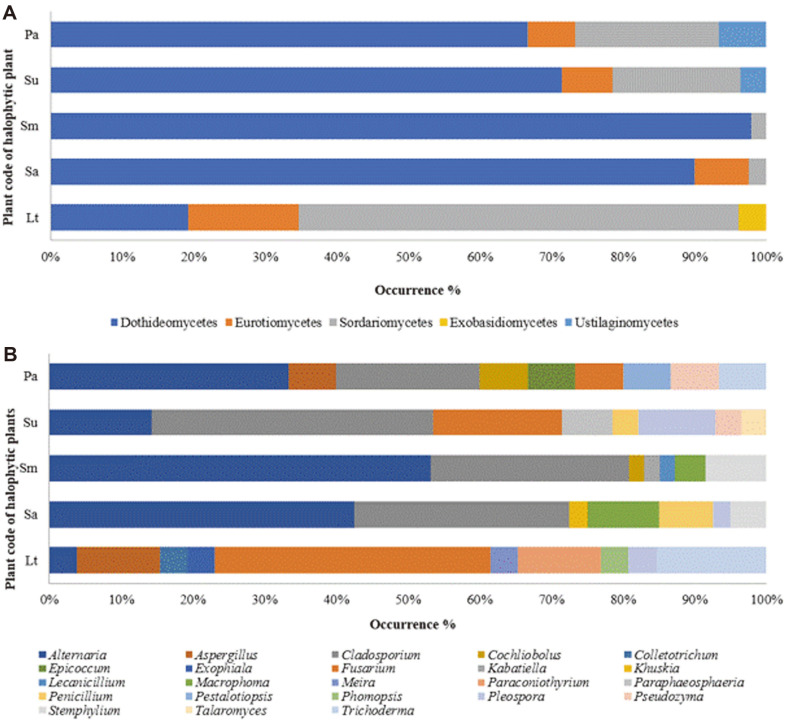
Distribution of the fungal isolates in different plant samples at the class (A) and genus (B) levels. *Lt, Limonium tetragonum; Sa, Suaeda australis; Sm, Suaeda maritima; Su, Suaeda glauca Bunge; and Pa, Phragmites australis*.

Taxonomic placement of endophytic fungi in each plant sample was analyzed at the class and genus levels (Fig. 1). Dothideomycetes from the Ascomycota phylum accounted for the highest percentage at the class level, except in the species *L. tetragonum*. Dothideomycetes accounted for more than half of the fungi in each plant sample. At the genus level, *Alternaria* (33.3%) was the most prominent, followed by *Cladosporium* (25%) in all of the plant samples except *S. glauca Bunge*, where *Cladosporium* was the most prominent genus followed by *Fusarium* (10.2%). In the plant samples of *L. tetragonum*, *Fusarium* was the most prevalent genus. *Alternaria* was found in all of the plant samples. The proportion of remaining fungal genera was approximately 0.5~2.5%. A previous study [26, 27] reported that the genus *Alternaria* was the most prominent fungus found in the roots of halophytic plants native to the Buan salt marsh. Fungal species, including those of the genera *Fusarium*, *Penicillium*, *Alternaria*, and *Cladosporium*, which are abundant in the host, occasionally establish endophytic associations with plants [34-38].

The current study isolated 156 fungal endophytes from salt-tolerant plants that were identified at the genus level and included *Alternaria*, *Aspergillus*, *Cladosporium*, *Cochliobolus*, *Colletotrichum*, *Epicoccum*, *Exophiala*, *Fusarium*, *Kabatiella*, *Khuskia*, *Lecanicillium*, *Macrophoma*, *Meira*, *Paraconiothyrium*, *Paraphaeosphaeria*, *Penicillium*, *Pestalotiopsis*, *Phomopsis*, *Pleospora*, *Pseudozyma*, *Stemphylium*, *Talaromyces*, and *Trichoderma*. Among the endophytic fungi isolated, the genera *Alternaria*, *Cladosporium*, and *Fusarium* were the most abundantly distributed in the plant samples. The phyla of various endophytic fungi were confirmed to be Ascomycota and Basidiomycota. These findings revealed that the roots of the plants growing in the coastal salt marsh are inhabited by diverse endophytic mycobiota.

Molecular techniques and sequencing of the genomic DNA are used widely and successfully for fungal identification [39]. DNA sequence analysis methods provided important information for this study. Most of the ribosomal DNA genes were highly conserved taxonomically for identification. The internal transcribed spacer (ITS) region has been commonly used as a DNA barcode for the molecular identification of fungi. This study was conducted with the 5.8S gene and ITS1/2 regions for the identification of the fungal strains [35].

### Diversity of the Endophytic Fungi Isolated from Halophytes

According to the host plant specimen results, the fungal isolates comprised 10 genera and 12 species isolated from *L. tetragonum*, 7 genera and 9 species from *S. australis*, 7 genera and 7 species from *S. maritima*, 8 genera and 6 species from *S. glauca Bunge*, and 9 genera and 6 species from *P. australis* (Table 2).

Generic richness and diversity were determined by counting the genera present in the fungal communities among the plant samples (Table 3). *P. australis* exhibited high scores compared to the other plant species in terms of both genus richness and diversity. Statistical analysis of richness demonstrated that *P. australis* had a Margalef ’s index of 2.95 and a Menhinick’s index of 2.32. The analysis of genetic diversity revealed a Fisher’s *α* index of 9.50, a Simpson’s index of diversity of 0.88, and Shannon’s index of 1.95. Thus, the Shannon index was less sensitive to evenness than the Fisher’s *α* and Simpson’s indices [40]. *P. australis* also exhibited the highest diversity index, as the fungi isolated from this plant were the most diverse compared to the other plant specimens. Statistical analysis of the endophytic fungi via counting the genera from the plant samples revealed that *P. australis* possessed the most diverse type of endophytic fungi. [15].

Fungal symbionts associated with plants in natural ecosystems help plants overcome abiotic stress, such as soil salinity, drought, and heat [41]. Shoreline habitats are frequently exposed to high salt stress, as they remain rhythmically submerged in saltwater. The capacity to resist high salinity stress is necessary for survival in coastal environments. Thus, fungal strains such us *Penicillium citrinum* and *Fusarium oxysporum* could improve the survival and growth of their hosts by enhancing tolerance to environmental stress [10, 11, 42, 43]. Many species of plant symbiotic fungi are known to produce a number of phytohormones [21, 22, 37, 38]. *Aspergillus* terreus and *P. citrinum* GAs that confer biotic stress resistance to pathogenic attack [44]. Plant hormones like GAs are essential for many developmental processes in plants, including seed germination, stem elongation, leaf expansion, ripening, and the induction of flowering [45]. Many researchers have found that fungal endophytes could limit the damage caused by pathogenic microorganisms and protect the host from diseases [12, 46, 47]. Moreover, it has been reported that *Penicillium* species increased salt stress resistance in the host plant [48, 49].

*Alternaria alternata* was the most abundant fungus among the collected samples and was isolated from plants *S. australis*, *S. maritima*, *S. glauca Bunge*, and *P. australis*. Previous reports have confirmed that *A. alternata* produces the plant growth regulator indole-acetic acid for the host plant [44] Indole-acetic acids are considered essential for important physiological processes such as cell division or cell elongation, tissue differentiation, phototrophic or geotropic responses, and all subsequent effects on plant growth and development [50]. Moreover, *Trichoderma harzianum* and *F. oxysporum* could produce indole-acetic acid [4, 51, 52]. Thus, these fungal species helped their host plants procure nutrients and promoted growth.

### Screening for Plant Growth-Promoting Effects of Fungal Culture Filtrates on Waito-C Rice Seedlings

Waito-C seeds were treated with uniconazole as a GA biosynthesis inhibitor. The Sm-3-7-5 fungal strain, which has plant growth-promoting capacity, was analyzed using the Waito-C rice seedlings. Screening of the microbial culture filtrates was used to identify the biologically active molecules to confirm the presence of gibberellins. In this study, plant growth-promoting hormones were detected in the culture filtrates of the fungal endophytes isolated from the roots of the halophytes using the Waito-C rice seedlings. Waito-C rice is a known dwarf rice cultivar with reduced GA biosynthesis [7, 18].

**Fig. 2:**
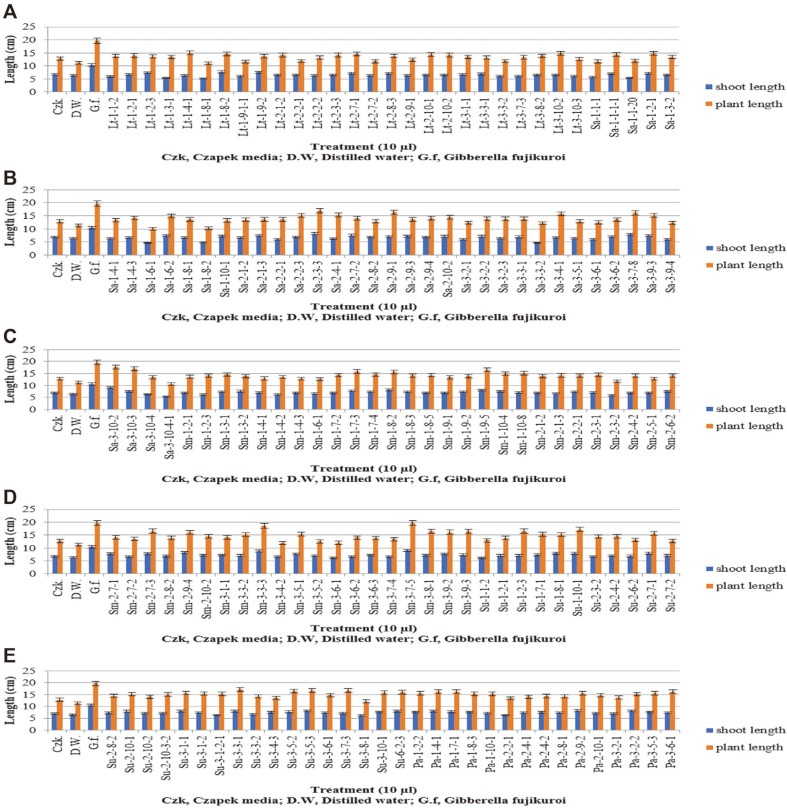
Waito-C rice seedlings with fungal culture filtrates of fungal endophytes plant growth promotion results (A, B, C, D, E). Ten microliters of lyophilized culture filtrates were applied to the Waito-C rice seedlings. The shoot length and plant length of the Waito-C rice seedlings were measured following 7 days of treatment. The standard deviation from the means was calculated using Microsoft Excel.

The culture filtrates of all of the fungal endophytes were applied to the Waito-C rice seedlings to analyze the plant growth promoting capacity (Fig. 2). The culture filtrate treatments using the Sm-3-7-5 fungal strain resulted in a 9.3 cm shoot length and 19.6 cm plant length as plant growth-promoting effects. Treatments with Sm-3-7-5 fungal strain or the *G. fujikuroi* strain revealed marked similarity in plant growth promotion between the two strains. These results were consistent with those of a previous study in which the endophytic fungus *Aspergillus clavatus* Y2H0002, isolated from the roots of *Nymphoides peltata*, was shown to promote the growth of various rice plants [53].

### Quantitative Analysis of Culture Filtrate of Sm-3-7-5 for the Presence of Gibberellins

In an analysis of growth promoting effect, Sm-3-7-5 produced the most effective growth promotion results. Comparing with *G. fujikuroi*, the two strains showed quite similar results in Waito-C growth. Because of that, additional comparison between the two strains was necessary. As GC/MS with selected ion monitoring technique has the ability to analyze highly-complex mixtures and detect compounds of different classes [54], it was used to analyze the culture filtrate of Sm-3-7-5 fungal strain. GC/MS SIM was important for the investigation of a number of compounds and was often used in plant experimentation [55, 56]. We also employed GC-MS SIM in the quantitative analysis of various plant hormones.

**Fig. 3:**
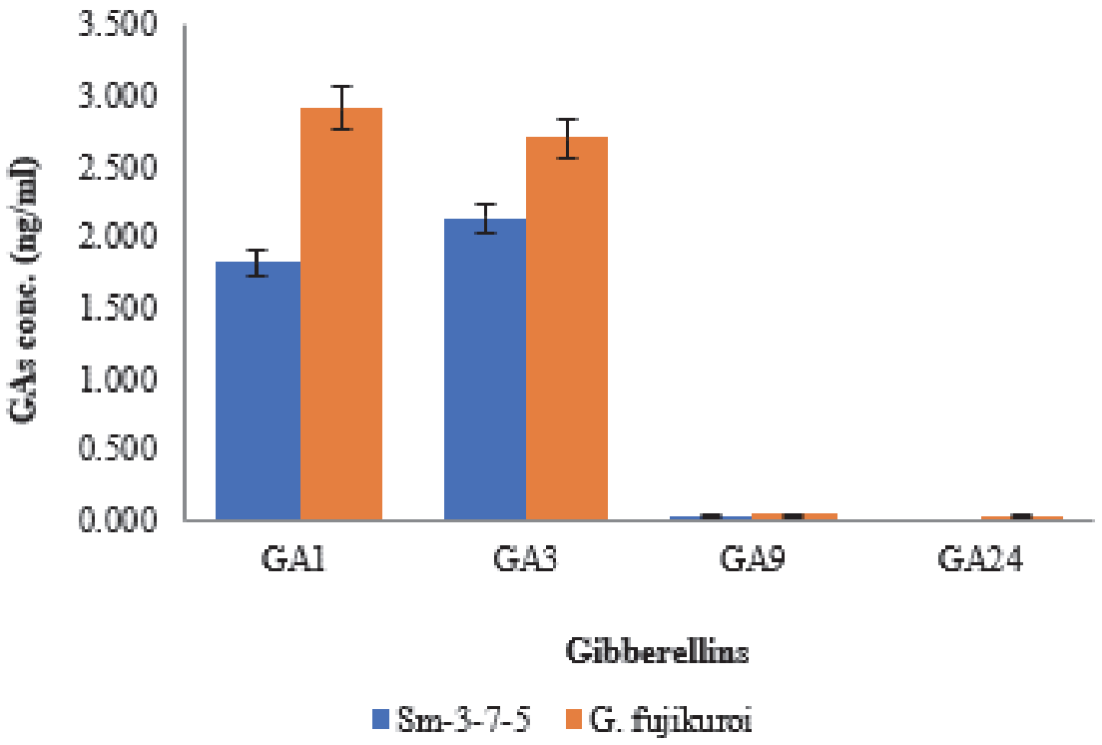
Gibberellins content of the fungal culture filtrates of the Sm-3-7-5 strain and wild type *Gibberella fujikuroi*. GC-MS SIM analysis of the culture filtrate extracts from the Sm-3-7-5 fungal strain detected two bioactive GAs. Sm-3-7-5 showed the presence of bioactivity of GA_1_, GA_3_, and other inactive GAs. The standard deviation from the means was calculated using Microsoft Excel.

Using HPLC and GC-MS, we analyzed gibberellins produced by fungal endophytes isolated from salt-tolerant plants. A variety of gibberellins were confirmed from the culture filtrate of the Sm-3-7-5 fungal strain; the results of the GC-MS SIM analysis showed that Sm-3-7-5 produced GA_1_ (1.820 ng/ml), GA_3_ (2.134 ng/ml), and other inactive GA_9_ (0.038 ng/ml) (Fig. 3). We confirmed that Sm-3-7-5 produced as much GA_1_, GA_3_, and GA_9_ as *G. fujikuroi*.

In summary, 156 strains of endophytic fungi were isolated from the roots of five halophytic plant species native to the Gochang salt marsh. These halophytes included *L. tetragonum*, *S. australis*, *S. maritima*, *S. glauca Bunge*, and *P. australis*. A study of the plant growth-promoting effects of *A. alternata* Sm-3-7-5 and GA production was conducted. All fungal strains were identified by molecular methods and were classified into 2 phyla, 5 classes, 11 orders, 15 families, and 23 genera. *Alternaria* and *Cladosporium* were found to be the dominant genera among the collected isolates. The most diverse group of fungi determined by the diversity analysis was isolated from the roots of *P. australis*. In conclusion, this study provides key information to the literature toward understanding the interactions between coastal plants and fungi.

## Figures and Tables

**Table 1:** Geographic coordinates and scientific names of the plants native to the Gochang salt marsh.

No.	Plant name	Plant code	Site of collection	Habitat
1	*Limonium tetragonum*	*Lt*	N 35°31′52.48′′/E 126°31′52.04′′	Halophytic
2	*Suaeda australis*	*Sa*	N 35°32′1.87′′/E 126°32′6.37′′	Halophytic
3	*Suaeda maritima*	*Sm*	N 35°32′2.16′′/E 126°32′5.90′′	Halophytic
4	*Suaeda glauca Bunge*	*Su*	N 35°32′1.87′′/E 126°32′7.45′′	Halophytic
5	*Phragmites australis*	*Pa*	N 35°32′2.43′′/E 126°32′6.05′′	Halophytic

**Table 2:** Endophytic fungi (156 strains) isolated from five coastal plants with scientific names, plant codes, taxa of fungal strain, and the number of fungal isolates.

Scientific name of plant sample	Abbreviated plant name	Taxa of fungal strains	No. of isolates
*Limonium tetragonum*	*Lt*	10 genera, 12 species	26
*Suaeda australis*	*Sa*	7 genera, 9 species	40
*Suaeda maritima*	*Sm*	7 genera, 7 species	47
*Suaeda glauca Bunge*	*Su*	8 genera, 6 species	28
*Phragmites australis*	*Pa*	9 genera, 6 species	15

*Lt*, *Limonium tetragonum*; *Sa*, *Suaeda australis*; *Sm*, *Suaeda maritime*; *Su*, *Suaeda glauca Bunge*; and *Pa*, *Phragmites australis*.

**Table 3:** Diversity indices and distribution of the endophytic fungi isolated from plants native to the Gochang salt marsh.

Fungal taxon	Lt	Sa	Sm	Su	Pa
*Alternaria*	1	17	25	4	5
*Aspergillus*	3				1
*Cladosporium*		12	13	11	3
*Cochliobolus*			1		1
*Colletotrichum*	1				
*Epicoccum*					1
*Exophiala*	1				
*Fusarium*	10			5	1
*Kabatiella*			1		
*Khuskia*		1			
*Lecanicillium*			1		
*Macrophoma*		4	2		
*Meira*	1				
*Paraconiothyrium*	3				
*Paraphaeosphaeria*				2	
*Penicillium*		3		1	
*Pestalotiopsis*					1
*Phomopsis*	1				
*Pleospora*	1	1		3	
*Pseudozyma*				1	1
*Stemphylium*		2	4		
*Talaromyces*				1	
*Trichoderma*	4				1

N	26	40	47	28	15
S	10	7	7	8	9

Shannon diversity index (*H'*)	1.91	1.48	1.28	1.74	**1.95**
Simpson’s index of diversity (*1-D*)	0.82	0.73	0.64	0.80	**0.88**
Menhinick’s index (*Dmn*)	1.96	1.11	1.02	1.51	**2.32**
Margalef ’s index (*Dmg*)	2.76	1.63	1.56	2.10	**2.95**
Fisher’s diversity (*α*)	5.95	2.46	2.28	3.74	**9.50**

*Lt*, *Limonium tetragonum*; *Sa*, *Suaeda australis*; *Sm*, *Suaeda maritime*; *Su*, *Suaeda glauca Bunge*; and *Pa*, *Phragmites australis*.
